# Case report: Accidental aconitine poisoning caused by the inappropriate use of a type of Chinese patent medicine

**DOI:** 10.3389/fphar.2024.1426006

**Published:** 2025-01-09

**Authors:** Ruikai Shang, Hongyu Liu, Qiaoxin Tian, Yuru Liu, Xiangdong Jian, Qilu Li

**Affiliations:** ^1^ Department of Occupational and Environmental Health, School of Public Health, Cheeloo College of Medicine, Shandong University, Jinan, Shandong, China; ^2^ Department of Poisoning and Occupational Diseases, Emergency Medicine, Cheeloo College of Medicine, Qilu Hospital of Shandong University, Shandong University, Jinan, Shandong, China; ^3^ School of Nursing and Rehabilitation, Cheeloo College of Medicine, Shandong University, Jinan, Shandong, China; ^4^ Department of Pharmacy, Cheeloo College of Medicine, The Hospital of Shandong University, Shandong University, Jinan, Shandong, China

**Keywords:** aconitine poisoning, arrhythmia, accident, Chinese patent medicine, treatment

## Abstract

**Background:**

Compound schizonepeta fumigation lotion is a type of Chinese patent medicine for external use. It has the effect of dispelling wind, eliminating dampness, reducing swelling, and relieving pain. Clinically, it is used for anal fumigation and treatment of external hemorrhoids, anal fissures, and other diseases. Aconitum species are widely used in the field of traditional Chinese medicine. However, improper use and overdose can easily cause acute poisoning, leading to malignant arrhythmias, cardiogenic shock, and even death.

**Methods:**

This study retrospectively analyzed the clinical data of eight patients who were treated in our hospital from June 2023 to February 2024 after taking compound schizonepeta fumigation lotion by mistake.

**Case presentation:**

We report 8 cases of patients who took compound schizonepeta fumigation lotion by mistake. Most patients were illiterate and older, and poisoning was attributed to the misuse of an external drug. The affected patients exhibited different degrees of arrhythmia, with 2 receiving tracheal intubation, assisted ventilation, and hemoperfusion. Finally, all patients were clinically cured and subsequently discharged.

**Conclusion:**

A considerable number of accidental aconitine poisoning cases were reported over a short period, alerting clinicians to their growing incidence. Meanwhile, when prescribing medications, clinicians must provide clear instructions on proper usage and ensure that patients strictly adhere to the dosage and administration guidelines outlined in the drug instructions. Additionally, patients should be informed that any errors in medication administration require urgent medical attention. Health professionals should aim to improve public understanding of safe medication practices, promote health literacy.

## 1 Introduction

Aconitine is a toxic alkaloid component of aconitum plants (Ranunculaceae), such as Radix Aconiti. Aconitum is widely used in the field of traditional Chinese medicine and is known to exert anti-inflammatory, analgesic, anti-tumor, and immune regulatory effects. Poisoning is frequently caused by excessive drug intake and improper drug administration. Compound schizonepeta fumigation lotion is composed of medicinal herbs such as Shengchuan Aconitum and Shengcao Aconitum and is prepared for external use. Clinically, it is used for anal fumigation and washing to treat external hemorrhoids and anal fissures. Many illiterate older adults misidentify a topical preparation for an oral preparation, which leads to poisoning. Arrhythmia is one of the most common clinical manifestations of aconitine poisoning, and even malignant arrhythmia has been documented in patients with severe poisoning (Liao et al., 2022). Herein, we report eight cases of accidental aconitine poisoning that occurred due to the inappropriate use of a Chinese patent medicine called compound schizonepeta fumigation lotion.

## 2 Case presentation

**FIGURE 1:**
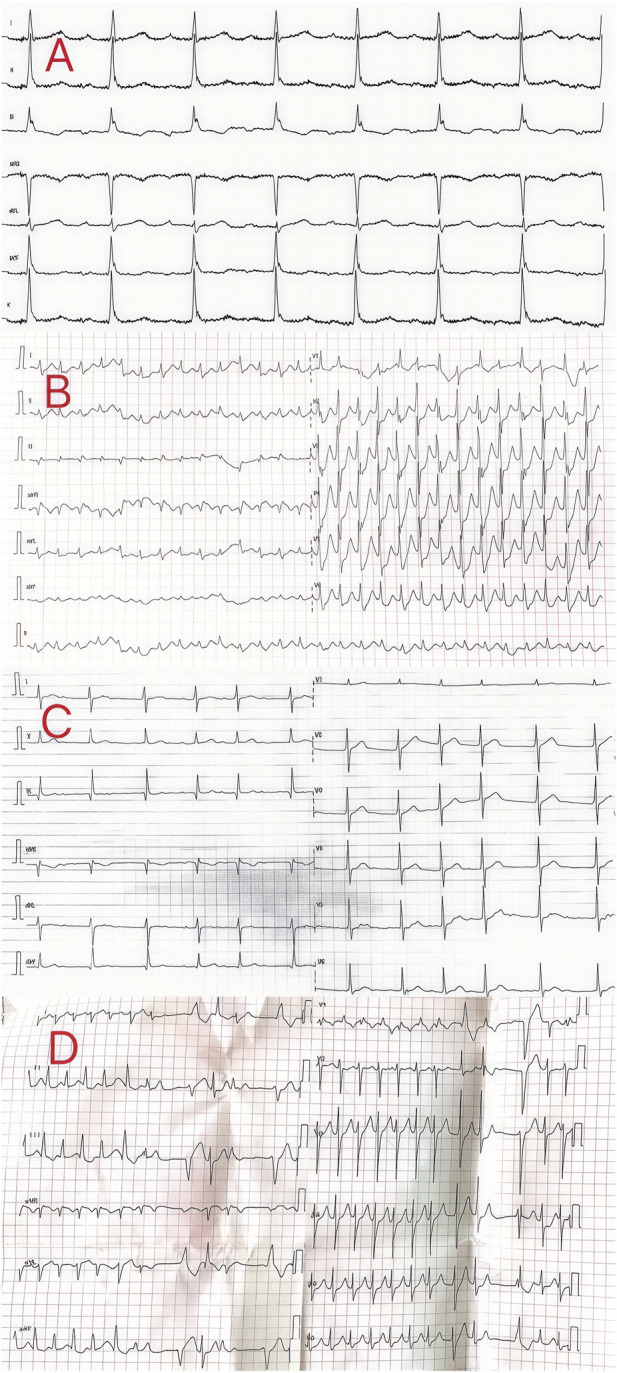
Electrocardiogram of the patients in cases 1–4.

Patient 1 was a 48-year-old man who ingested 10 g of compound schizonepeta fumigation lotion on 30 July 2023. After ingestion, he experienced nausea and vomiting, accompanied by limb numbness, lower limb weakness, heart discomfort, and a burning sensation. For further diagnosis and treatment, he was immediately transferred to our hospital at 23:56 p.m. (the day of ingestion). Physical examination on admission revealed a body temperature of 37.1°C, heart rate of 113 beats/min, respiration rate of 17 breaths/min, blood pressure of 65/31 mmHg, clear consciousness, and depressed mood. His heart sounds were vigorous and irregular, and no pathological murmurs were heard in any of the valve regions. An electrocardiogram on admission showed a sinus rhythm with an atrioventricular block and inferior wall T wave abnormality (Figure 1A). Laboratory tests revealed no obvious abnormalities in routine blood parameters, myocardial enzymes, coagulation functions, and liver and kidney functions.

The patient had a blood aconitine concentration of 48 ng/mL; hence, a diagnosis of aconitine poisoning was established. He underwent electrocardiographic monitoring and received activated charcoal, montmorillonite powder, and mannitol for catharsis and detoxification. Additionally, oxygen supplementation, fluid infusion, anti-shock measures, diuresis, and other symptomatic treatments were administered. Lidocaine was also used to correct the arrhythmias. The following day, the electrocardiogram showed that the patient’s heart rhythm had returned to a normal sinus rhythm. His symptoms of nausea, vomiting, limb numbness, and general weakness showed substantial improvement when compared with those recorded on the previous day, and his condition remained stable. Echocardiogram revealed mild aortic and tricuspid regurgitation. The patient was discharged after 5 days. At a follow-up visit, all the indicators were normal.

Patient 2 was a 72-year-old woman who ingested 10 g of compound schizonepeta fumigation lotion on 13 June 2023. After ingestion, she presented with vomiting, diarrhea, and palpitations. For further diagnosis and treatment, she was transferred to our hospital on the following day. Physical examination on admission revealed a body temperature of 37°C, heart rate of 172 beats/min, respiration rate of 16 breaths/min, blood pressure of 64/42 mmHg, clear consciousness, and depressed mood. An electrocardiogram conducted on the day of admission showed supraventricular tachycardia and ST-T abnormalities in the anterior/septal and lateral walls (Figure 1B). Laboratory test results were as follows: serum myoglobin, 165.40 ng/mL (0–70 ng/mL); creatine kinase isoenzyme, 6.50 ng/mL (0.3–4 ng/mL); serum high-sensitivity troponin, 75.97 ng/L (<17.5 ng/L); total protein (dry), 60 g/L (63–82 g/L); urea nitrogen, 6.8 mmol/L (2.5–6.1 mmol/L). Routine blood tests and coagulation functions were normal. The admission diagnosis was aconitine poisoning. The patient underwent electrocardiographic monitoring, which showed that the heart rate waveform was disordered, the blood pressure was markedly low, and the patient was in a shocked state. Treatment primarily included symptomatic treatment and correction of the shock, maintenance of electrolyte balance, essential organ protection, such as the liver and kidneys, careful monitoring of the balance of intake and output, and performing timely electrocardiographic and related indicator reassessments. An electrocardiogram performed the following day showed that the patient’s heart rhythm had returned to a normal sinus rhythm, and her condition improved with stable signs. The patient requested to be discharged and consented to the decision. After follow-up, the patient recovered fully and was living a normal life.

Patient 3 was a 33-year-old man who ingested 10 g of compound schizonepeta fumigation lotion on 15 October 2023. After ingestion, he experienced dizziness, oral numbness, chest tightness, nausea, and vomiting. For further diagnosis and treatment, he was transferred to our hospital on the following day. Physical examination on admission revealed a body temperature of 37.3°C, heart rate of 80 beats/min, respiration rate of 17 breaths/min, and blood pressure of 119/76 mmHg. Moreover, the patient presented with a clear consciousness and was in good spirits. An electrocardiogram conducted on the day of admission revealed atrial fibrillation with mild right axis deviation (Figure 1C). Laboratory tests showed no obvious abnormalities in routine blood parameters, myocardial enzymes, coagulation functions, and liver and kidney functions. The admission diagnosis was aconitine poisoning. The patient underwent electrocardiographic monitoring and received activated charcoal, montmorillonite powder, and mannitol for catharsis and detoxification. Additionally, oxygen supplementation, organ protection, fluid replacement, nutritional support, and other comprehensive treatments, along with dynamic observation of changes in his condition, were performed. An electrocardiogram conducted in the afternoon of the same day showed that the patient’s heart rhythm had returned to a normal sinus rhythm, and he was cured and discharged 2 days later. After follow-up, the patient recovered fully and was living a normal life.

Patient 4 was a 53-year-old woman who ingested 20 g of compound schizonepeta fumigation lotion on 27 January 2024. After ingesting the compound, she developed an arrhythmia and exhibited a progressive decrease in blood oxygen saturation; symptomatic treatments such as tracheal intubation, assisted ventilation, and drug control of her ventricular rate were provided. An electrocardiogram conducted at the local hospital revealed a rapid ventricular rate, atrial fibrillation with ventricular premature beats, an incomplete right bundle branch block, and moderate ST depression (Figure 1D). She was transferred to our hospital on the same day. Physical examination on admission revealed a body temperature of 36.3°C, heart rate of 89 beats/min, respiration rate of 16 breaths/min, and blood pressure of 147/93 mmHg. Additionally, the patient presented with symptoms of drowsiness, passive positioning, and lack of cooperation during physical examination. An electrocardiogram conducted at our hospital revealed a rapid ventricular rate, atrial fibrillation with premature ventricular contractions, incomplete right bundle branch block, and moderate ST depression. Laboratory tests revealed the following findings: neutrophil ratio, 90.40% (40%–75%); alanine aminotransferase level, 97 IU/L (0–35 IU/L); and aspartate aminotransferase level, 94 IU/L (14–36 IU/L). Measurement of aconitine, hypoaconitine, and mesoaconitine concentrations revealed blood levels of 42, 18, and 6 ng/mL, respectively. Accordingly, the admission diagnosis was aconitine poisoning. The patient underwent electrocardiographic monitoring and received activated charcoal, montmorillonite powder, and mannitol for catharsis and detoxification. Additionally, organ protection, prevention of infection, fluid replacement, nutritional support, hemoperfusion, and other comprehensive treatments were performed. On day 3 of admission, the patient’s liver and kidney functions and myocardial enzyme spectrum were normal. Subsequently, the patient’s orotracheal cannula was removed, and oxygen inhalation through a nasal catheter was administered while closely monitoring dynamic changes in her condition. On day 4 of admission, the blood concentrations of aconitine, hypoaconitine, and mesaconitine were re-examined and were undetectable. Additionally, the femoral vein catheter was removed. Seven days later, she was cured and discharged and scheduled for regular follow-up visits.

**FIGURE 2:**
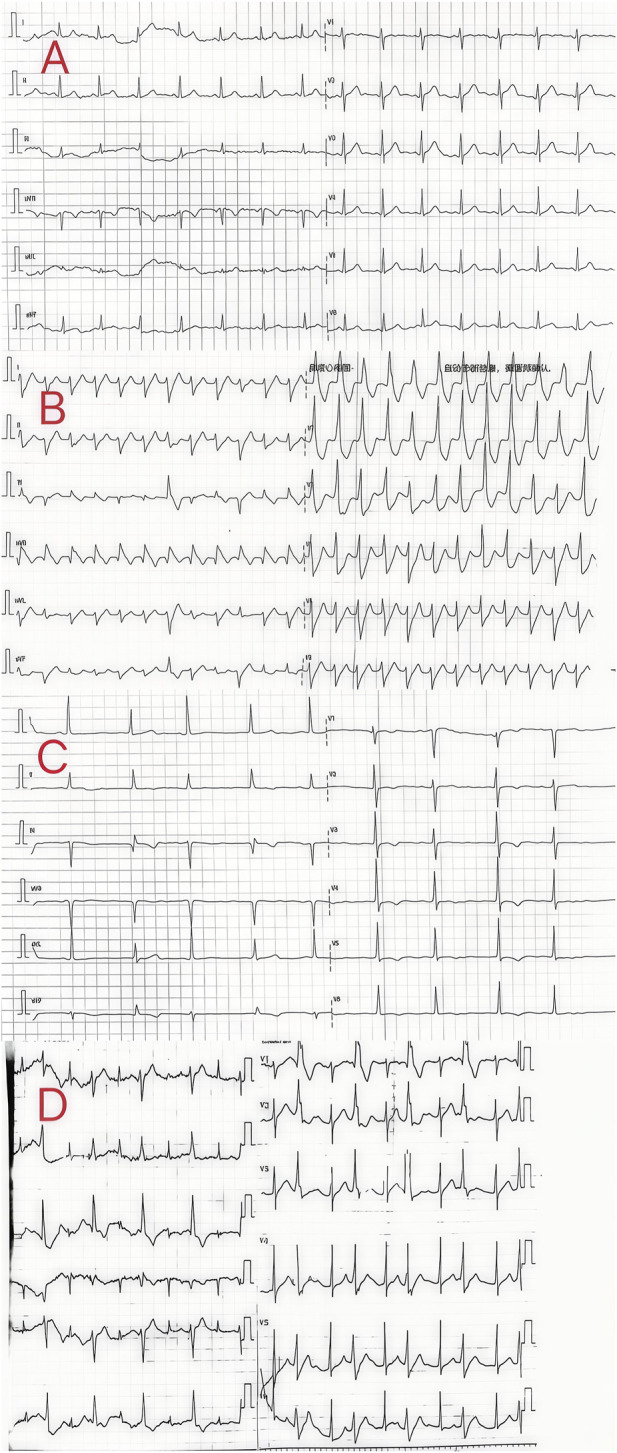
Electrocardiogram of the patients in cases 5–8.

Patient 5 was a 13-year-old woman who ingested 5 g of compound schizonepeta fumigation lotion on 30 January 2024. After ingestion, she presented with dizziness, hand numbness, heartburn, and other symptoms. For further diagnosis and treatment, she was transferred to our hospital on the following day. Physical examination on admission revealed a body temperature of 37.1°C, heart rate of 90 beats/min, respiration rate of 21 breaths/min, blood pressure of 130/69 mmHg, clear consciousness, and good mental state. The electrocardiogram revealed a sinus rhythm (Figure 2A). Laboratory tests showed no obvious abnormalities in routine blood parameters, myocardial enzymes, coagulation functions, and liver and kidney functions. The patient had blood aconitine and hypoaconitine concentrations of 12 and 7 ng/mL, respectively. The admission diagnosis was aconitine poisoning. The patient underwent electrocardiographic monitoring and received activated charcoal, montmorillonite powder, and mannitol for catharsis and detoxification. Additionally, organ protection, fluid replacement, nutritional support, and other comprehensive treatments were performed. A re-examination of liver and kidney functions and myocardial enzyme spectrum revealed normal findings. Seven days later, she was cured and discharged and scheduled for regular follow-up visits.

Patient 6 was a 60-year-old man who ingested 10 g of compound schizonepeta fumigation lotion on 4 February 2024. After ingestion, he presented with numbness in his limbs. For further diagnosis and treatment, he was transferred to our hospital on the same day. Physical examination on admission revealed a body temperature of 36.7°C, a heart rate of 130 beats/min, a respiration rate of 14 breaths/min, a blood pressure of 91/57 mmHg, clear consciousness, and good mental state. Although an irregular heartbeat was detected, there was no obvious murmur. The electrocardiogram on admission showed ventricular tachycardia (Figure 2B). Laboratory tests showed no obvious abnormalities in routine blood parameters, myocardial enzymes, coagulation functions, and liver and kidney functions. Measurement of aconitine, hypoaconitine, and mesoaconitine concentrations revealed blood levels of 39, 18, and 12 ng/mL, respectively. Accordingly, the admission diagnosis was aconitine poisoning. The patient underwent electrocardiographic monitoring and received activated charcoal, montmorillonite powder, and mannitol for catharsis and detoxification. Additionally, organ protection, prevention of infection, fluid replacement, nutritional support, and other comprehensive treatments were performed. The following day, the patient’s heart rhythm returned to a normal sinus rhythm, and the liver and kidney function and myocardial enzyme spectrum were normal. Five days later, he was cured and discharged. A telephonic follow-up was performed, indicating that bodily functions had returned to normal.

Patient 7 was a 73-year-old woman who ingested 10 g of compound schizonepeta fumigation lotion on 6 February 2024. She was transferred to our hospital on the same day after symptomatic treatment, including gastric lavage, at a local hospital. Physical examination on admission (at our hospital) revealed a body temperature of 36.9°C, heart rate of 66 beats/min, respiration rate of 16 breaths/min, blood pressure of 164/76 mmHg, clear consciousness, and sound mental alertness. The electrocardiogram on admission revealed a sinus rhythm and extensive T-wave abnormalities (Figure 2C). Laboratory tests revealed no obvious abnormalities in routine blood parameters, myocardial enzymes*,* coagulation functions, and liver and kidney functions. Measurement of aconitine, hypoaconitine, and mesoaconitine concentrations revealed blood levels of 16, 9, and 6 ng/mL, respectively. Accordingly, the admission diagnosis was aconitine poisoning. The patient underwent electrocardiographic monitoring and received activated charcoal, montmorillonite powder, and mannitol for catharsis and detoxification. Additionally, organ protection, fluid replacement, nutritional support, and other comprehensive treatments were performed. On day 3 of admission, the liver and kidney functions and myocardial enzymes were normal. She was cured and discharged 3 days later and scheduled for regular follow-up visits.

**FIGURE 3:**
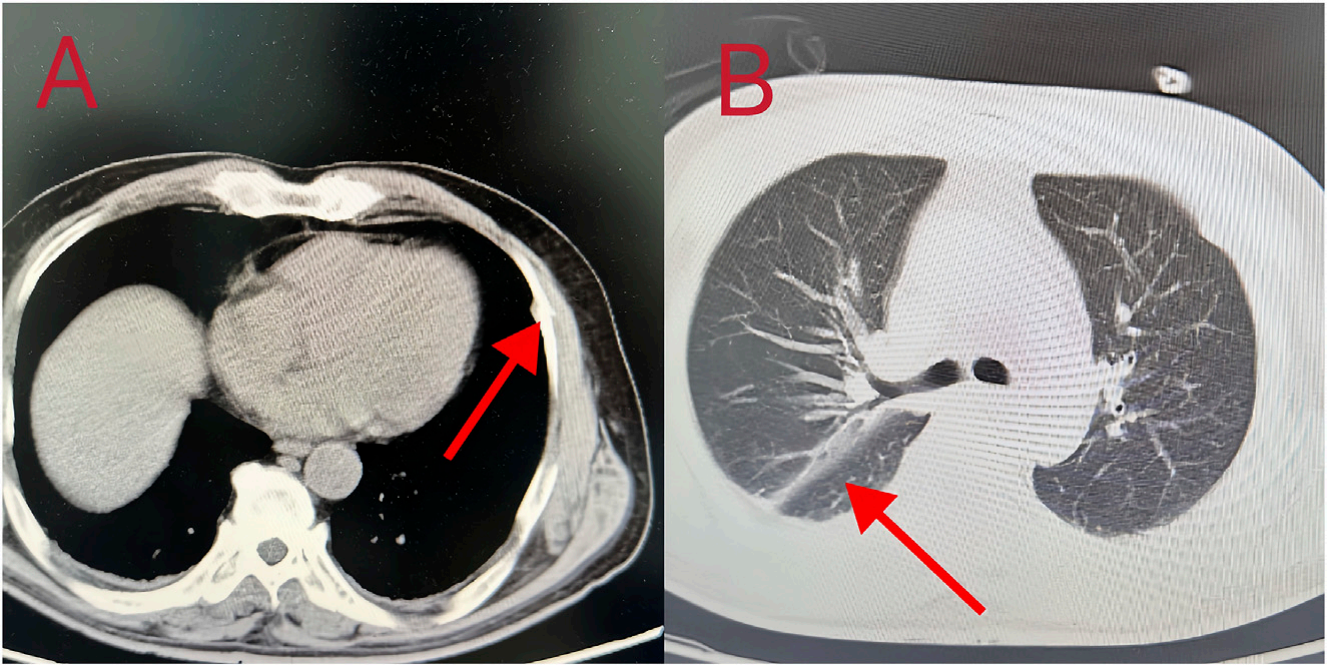
**(A, B)** Chest CT images showed bilateral pneumonia, a small amount of bilateral pleural effusion, and partial rib bone discontinuity.

**FIGURE 4:**
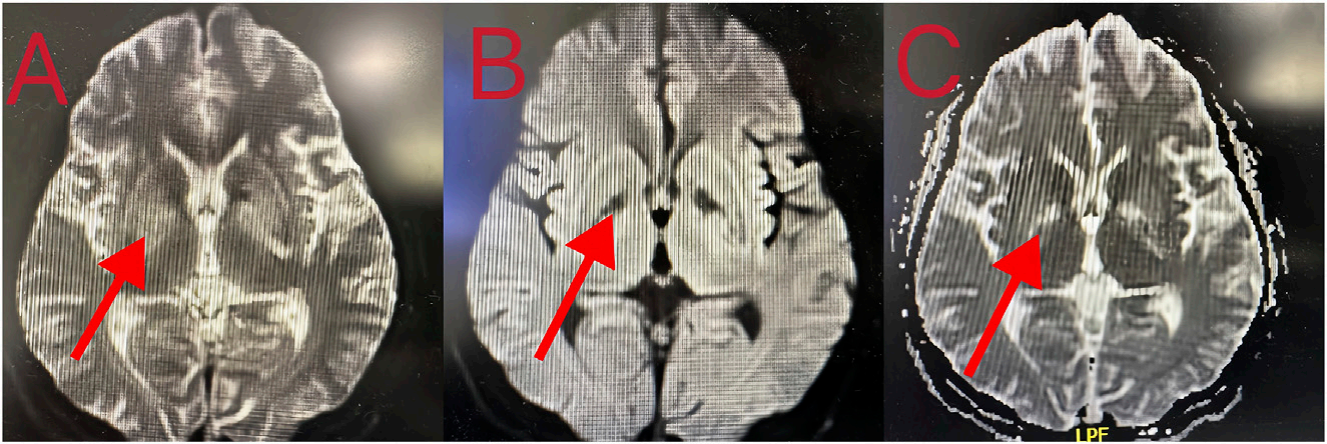
**(A–C)** Brain MRI showing that the density of nuclei in the bilateral basal ganglia was generally reduced, which was consistent with toxic encephalopathy.

Patient 8 was a 53-year-old man who ingested 10 g of compound schizonepeta fumigation lotion on 4 February 2024, and was admitted to a local hospital. An electrocardiogram conducted at the local hospital revealed ventricular tachycardia and premature ventricular contractions (Figure 2D). After admission, he continued to experience malignant arrhythmias and unstable circulation. He received extracorporeal membrane oxygenation supportive treatment for 4 days, tracheal intubation and assisted ventilation for 8 days, hemofiltration five times, and hemoperfusion five times. For further diagnosis and treatment, he was transferred to our hospital on 16 February 2024. Physical examination on admission (at our hospital) revealed a body temperature of 38.5°C, heart rate of 92 beats/min, respiration rate of 16 breaths/min, blood pressure of 135/82 mmHg, an impaired mental state, and an inability to communicate. The electrocardiogram upon admission revealed a sinus rhythm and non-specific T-wave abnormalities. Laboratory test results were as follows: white blood cell count, 16.50 × 10^9^/L (3.5–9.5 × 10^9^/L); neutrophil ratio, 77.50% (40%–75%); alanine aminotransferase, 61 IU/L (9–50 U/L); creatine kinase, 649 IU/L (55–170 IU/L); and creatine kinase isoenzyme, 4.30 ng/mL (0.3–4 ng/mL). At the local hospital, measurement of aconitine, hypoaconitine, and mesoaconitine concentrations revealed blood levels of 205, 58, and 23 ng/mL, respectively. However, upon transfer to our hospital, these compounds were untraceable in the patient’s blood. The admission diagnosis was aconitine poisoning. The patient underwent electrocardiographic monitoring, organ protection, anti-infective treatment, fluid replacement, nutritional support, and other comprehensive treatments. On day 3 of admission, his white blood cell count was 22.50 × 10^9^/L (3.5–9.5 × 10^9^/L), neutrophil ratio was 87% (40%–75%), creatine kinase level was 182 IU/L (55–170 IU/L), and liver and kidney functions were normal. Chest computed tomography revealed slight inflammation in both lungs and a partial left rib fracture (Figures 3A, B). Brain magnetic resonance imaging revealed symmetrical abnormal signals in bilateral basal ganglia and bilateral parietal and temporal cortices, which was considered to be due to toxic encephalopathy based on his medical history (Figures 4A–C). Salvianolate (200 mg qd) was given to improve microcirculation symptomatic treatment. On day 7 of admission, white blood cell count, 12.98 × 10^9^/L (3.5–9.5 × 10^9^/L), the liver and kidney functions and myocardial enzymes were normal. After 18 days of treatment, the patient improved and was discharged. The patient was discharged for local rehabilitation. During the recent telephonic follow-up, the patient basically returned to normal but still had stuttering and a low voice tone. The family members will bring the patient to the hospital for reexamination in the near future.

## 3 Discussion and conclusion

Compound schizonepeta fumigation lotion contains Chinese medicinal herbs such as Shengchuan aconitum and Shengcao aconitum, 10 g per bag (equivalent to 28 g of the original medicinal component). The main toxic component is aconitine, with a toxic dose of 0.2 mg and a lethal dose of 2–4 mg (Jeon et al., 2021). Predominant manifestations of aconitine poisoning include cardiovascular, digestive, and nervous system symptoms. Most patients initially experience perioral and facial numbness, nausea and vomiting, abdominal pain and diarrhea, and palpitations. Individuals experiencing severe poisoning may exhibit signs of coma, arrhythmia, circulatory and respiratory failure, and even death (Lin et al., 2004). Aconitine is mainly ingested orally, absorbed in the gastrointestinal tract, and metabolized by the liver and kidney (Fujita et al., 2007). Aconitine poisoning can cause several types of arrhythmia, even fatal malignant arrhythmia (Chan, 2009). Reported electrocardiogram findings were diverse (Table 1), but ventricular arrhythmia was the most common (Karturi et al., 2016). The mechanisms by which aconitine causes arrhythmias include prolonging the action potential duration by opening the sodium channel and non-selectively blocking the potassium channel. By affecting calcium ion channels, intracellular calcium overload and myocardial excitation and contraction coupling processes are affected, which leads to arrhythmia (Jiang et al., 2023). Aconitine can also stimulate the cardiac vagus nerve, reduce the automaticity and conductivity of the sinus node, partly cause conduction blocks or even arrest, and partially trigger abnormal excitation or reentry, all of which lead to arrhythmia (Sheth et al., 2015). In addition, aconitum alkaloids can directly act on cardiomyocytes by affecting mitochondria, Na^+^ channels, Ca^2+^ channels, K^+^ channels, and connexin 43 and causing apoptosis and oxidative damage in cardiomyocytes (Zhou et al., 2013; Sun et al., 2014; Chen et al., 2016; Zhang et al., 2007). Aconitum alkaloids can induce the abnormal secretion of neurotransmitters, such as γ-aminobutyric acid, thereby damaging the nervous system. Inhibition of cholinergic nerves can cause M- and N-like symptoms and act on the respiratory center, resulting in death (Peng et al., 2009).

**TABLE 1:** Clinical data of eight patients with acute aconitine poisoning.

Patient	Sex (F/M)	Age (y)	Oral dose (g)	Concentration of aconitine in the blood (ng/mL)	Time interval between oral administration and admission	Length of stay (d)	Key clinical diagnoses	Electrocardiographic findings	Prognosis
1	M	48	10	48	4 h	5	Aconitine poisoning	Sinus rhythm with atrioventricular block and inferior wall T wave abnormality	Recovered
2	F	72	10	-	5 h	2	Aconitine poisoning	Supraventricular tachycardia and ST-T abnormalities in anterior/septal and lateral walls	Recovered
3	M	33	10	-	4 h	2	Aconitine poisoning	Atrial fibrillation with mild right axis deviation	Recovered
4	F	53	20	42	7 h	7	Aconitine poisoning	Rapid ventricular rate, atrial fibrillation with ventricular premature beats, incomplete right bundle branch block, and moderate ST depression	Recovered
5	F	13	5	12	7 h	7	Aconitine poisoning	Sinus rhythm	Recovered
6	M	60	10	39	4 h	5	Aconitine poisoning	Ventricular tachycardia	Recovered
7	F	73	10	16	5 h	3	Aconitine poisoning	Sinus rhythm and extensive T-wave abnormalities	Recovered
8	M	53	10	205	13 days	18	Aconitine poisoning	Ventricular tachycardia and premature ventricular contractions	Recovered

All patients in this report suffered from hemorrhoids and were prescribed compound schizonepeta fumigation lotion for external use in the hospital. However, all patients took the compound schizonepeta fumigation lotion orally, which was confirmed by the presence of aconitine components in all blood samples. This occurred because most of the patients were illiterate or older, meaning they could not effectively read the instructions, and the packaging was similar to that for oral powder, leading to confusion and poisoning. All patients with poisoning had evident arrhythmia in the early stage, and one patient even had cardiac arrest. Cardiopulmonary resuscitation was performed and extracorporeal membrane oxygenation was provided. We searched PubMed and CNKI databases, but only three relevant articles (Zhao et al., 2011; Lin and Hao, 2024; Cao et al., 2024) were found. Combined with this report, it was found that aconitine poisoning events caused by the improper use of compound schizonepeta fumigation lotion occurred occasionally, mostly in adolescents and older people, with a low mortality rate and good overall prognosis (Table 2). The possible reason is that most patients were aware of the mistake immediately upon consumption and were treated in time. Although all patients were cured after active treatment, care should be taken to avoid administering compound schizonepeta fumigation lotion orally in clinical practice. Electrocardiogram monitoring, thorough gastric lavage, large amount of fluid infusion, and diuresis should be performed immediately. Blood purification should be performed if necessary, and possible electrolyte disorders should be corrected. When arrhythmia occurred, active antiarrhythmic treatment was provided (Coulson et al., 2017). This type of poisoning may cause malignant arrhythmia or even cardiac arrest (case 8).

**TABLE 2:** Literature case summary.

Case and reference	Sex	Age	Dose (g)	Main clinical manifestations	Prognosis
1 (Zhao et al., 2011)	F	16	10	Initially, she felt numbness in the mouth and face and then in the limbs, with dizziness, chest tightness, palpitation, and irritability	Recovered
2 (Zhao et al., 2011)	F	18	10	Numbness of the lips and face followed by numbness of the limbs, burning pain in the throat, dizziness, chest tightness, palpitation, and restlessness	Recovered
3 (Zhao et al., 2011)	M	18	10	Numbness of limbs, dizziness, chest tightness, palpitation, and restlessness	Recovered
4 (Zhao et al., 2011)	M	19	10	At the time of admission, he was unconscious, accompanied by limb convulsions. Electrocardiogram monitoring showed sinus tachycardia and low blood pressure	Recovered
5 (Lin and Hao, 2024)	M	73	10	He developed vomiting and dizziness 10 min after taking the medicine and became confused on the way to the hospital. He became unconscious after entering the emergency department	Recovered
6 (Cao et al., 2024)	M	74	5	Abdominal distension, dizziness, nausea, and numbness of both upper limbs	Recovered

In summary, a considerable number of accidental aconitine poisonings occurred over a short period, alerting clinicians. Therefore, when prescribing medications, clinicians must provide clear instructions on proper usage and ensure that patients strictly adhere to the dosage and administration guidelines outlined in the drug instructions. Additionally, patients should be informed that any errors in medication administration require urgent medical attention. Health professionals should aim to improve public understanding of safe medication practices, promote health literacy, and make sure that patients fully understand any instructions for self-medication. For older people, their sensory function and cognitive ability are weakened, and there are often hidden dangers in medication. Therefore, family members of patients should be involved in managing and monitoring their medication.

## Data Availability

The original contributions presented in the study are included in the article/supplementary material, further inquiries can be directed to the corresponding author.
